# Rheological properties of wood/bacterial cellulose and chitin nano‐hydrogels as a function of concentration and their nano‐films properties

**DOI:** 10.1049/nbt2.12083

**Published:** 2022-04-04

**Authors:** Hesamoddin Jannatamani, Ali Motamedzadegan, Mohammad Farsi, Hossein Yousefi

**Affiliations:** ^1^ Department of Food Science and Technology Management Islamic Azad University Sari Branch Sari Iran; ^2^ Department of Food Science and Technology Sari Agricultural Sciences and Natural Resources University Moji Iran; ^3^ Laboratory of Sustainable Nanomaterials, Department of Wood Engineering and Technology Gorgan University of Agricultural Sciences and Natural Resources Gorgan Iran

**Keywords:** bacterial cellulose nanofiber, chitin nanofiber, nano‐hydrogel, rheology, wood cellulose nanofiber

## Abstract

In this study, rheological properties of the Wood Cellulose NanoFibers (WCNF), Bacterial Cellulose NanoFibers (BCNF), and Chitin NanoFibers (ChNF) as well as physical properties of films prepared from each nano‐hydrogel were investigated. Each nano‐hydrogel was prepared in 2 concentrations of 0.5 and 1 wt% for rheological study. Rheological properties were measured using a rotational rheometer. The flow behaviour data were fitted with rheological models. Apparent viscosity was higher in higher concentrations of nano‐hydrogels. Herschel‐Bulkley model was the best model for flow behaviour data fitting. BCNF nano‐hydrogels had the highest hysteresis loop while WCNF nano‐hydrogels had the best structure recovery and lowest hysteresis loop. At LVE (Linear Viscoelastic Region), G′ (storage modulus) and G″ (loss modulus) had a constant value, but as strain increased their values decreased. Storage modulus was found to be greater than loss modulus in all samples during frequency sweep test. BCNF nano‐hydrogel showed the lowest frequency dependency. Chitin nanofilms had the highest elongation and stress value.

## 1 INTRODUCTION

Cellulose and chitin are structural polymers predominantly found in plants and aquatic animals. In recent decades, people have shown a tendency towards consumption of the products produced from renewable‐based resources. Cellulose and chitin, the most plentiful biopolymers on the Earth are environmentally friendly, biodegradable, and non‐toxic materials with a wide‐speared use in several areas such as food and cosmetic industrial products, biomedical, filtration, and packaging sectors [1]. Cellulose nanofiber (CNF) can be obtained from lignocellulosic sources such as wood and agricultural residues. On the other hand, with cellulose, nanocellulose fibres have higher active hydroxyl groups as well as higher surface area ratio [2]. High density of hydroxyl groups is one of the excellent features of cellulose‐based compounds leading to its hydrophilic nature and make it a good candidate for production of nano‐hydrogels [3]. Nano‐hydrogels are macromolecular networks absorbing and desorbing the aqueous solutions in response to environmental stimulus. The amount of water trapped in nano‐hydrogel network depends on the polymeric network and environmental conditions such as temperature, pH, and ionic strength of the aqueous solution [2]. Cellulose nanofibers can be considered as functional materials because of their unique features like being individualised, continuous, as well as having constant thickness, and a high crystallinity [3]. Chitin nanofibers (ChNF) are mainly produced from shrimp and crab shells using some acidic and alkaline processes [4].

Nanocellulose can be produced by two different approaches: top‐down approach involving the mechanical, chemical or enzymatic degradation, and bottom‐up approach including bacterial cellulose synthesis [5], to be more exact, production of bacterial cellulose follows a way, in which building up of the bundles of nanofibrils is done by some bacterial species especially Acetobactor Xylinus in aqueous culture media during a couple of days up to 2 weeks [6]. Several hydroxyl groups present in the bacterial cellulose surface make it a hydrophilic material. Bacterial cellulose nanoFibers have a diameter of approximately 20–100 nm and display a superior surface area rather than vegetal cellulose [7]. Chitin individual nanoFibers have been shown to be produced by the downsizing process [8] .Chitin, the second abundant normal polymer after cellulose, is principally found inside the exoskeleton of crustaceans and furthermore the cell dividers of numerous insects. Chitin has widespread applications in water treatment, textile, medicine, pharmaceuticals, food, and agriculture due to its nontoxicity, biodegradability, biocompatibility, antimicrobial and antioxidant properties. Both nanochitin and nanocellulose are found either in crystalline or fibrous form. Nanofibers have aspect ratios higher than nanocrystals, but the crystallinity index of the nanofiber is less than that of nanocrystals [6] .The material strength depends on the intrinsic storage modulus of the NFs, the density of the crosslinking or the number of entanglement points, and the bonding between the NFs [9]. Agoda‐Tandjawa et al. [3], investigated the rheological properties of Micro‐fibrillated cellulose (MFC) suspensions. They studied the effect of some experimental parameters such as cellulose concentration, temperature, ionic strength, and pH on the rheological properties of cellulose. They showed that, cellulose suspensions at different concentrations (even at the lowest concentration) had a viscoelastic solid‐like behaviour. Suenaga and Osada [9], measured dynamic viscoelasticity of Chitin nanofiber (ChNF) dispersions with various concentrations, disintegration times, acidities, and crystalline structures. The ChNF dispersions showed elastic behaviour at 0.05 w/v% concentration. The storage modulus of the ChNF was greater than the nanorod and nanowhisker chitin solutions at the same solid concentrations.

Numata et al. [10], studied the structural and rheological properties of the fruits. The three bacteria isolated were identified including one strain of Gluconacetobacter sp. and two strains of Gluconacetobacter hansenii. They showed that the rheological properties of the BC pellicles were strongly influenced by the network structure of the cellulose fibres rather than fibre concentrations. Jiang et al. [11], studied on edible, and structured emulsion gel which was successfully prepared from thermo‐gelable polysaccharide curdlan and regenerated cellulose. The effect of temperature and RC/curdlan concentrations on the rheological behaviour of the emulsion gel was evaluated.

WCNF, BCNF and ChNF have high aspect ratio (length to width ratio). During downsizing process, the diameter of cellulose fibres decreased from about 30,000 nm to less than 50 nm. During this miniaturisation of size, the special surface area increased from about 3 to 5 m^2^/g to over 100 m^2^/g. High ratio of length to diameter, high degree of crystalline and existence of fibrillated structures make these nanostructures highly absorbent materials. In addition, they have usually the 3‐D network structure which provide high capillary forces to keep and absorb water inside the network. Because of these, WCNF, BCNF and ChNF can make the nano hydrogel. Considering numerous promising properties of cellulose and chitin and their wide variety of applications, it was aimed to study on viscosity and rheology of cellulose and chitin nano hydrogels and compare their results. Rheology is the study of flow and deformation of materials under applied forces which is routinely measured using a rheometer. The measurement of rheological properties is applicable to all materials—from fluids such as dilute solutions of polymers and surfactants to concentrated protein formulations, to semi‐solids such as pastes and creams, to molten or solid polymers as well as asphalt. Rheological properties can be measured from bulk sample deformation using a mechanical rheometer, or on a micro‐scale by using a microcapillary viscometer or an optical technique such as microrheology.

The present study was done to investigate the viscosity and rheological properties of cellulose and chitin nano‐hydrogels, and the morphological, chemical structure and mechanical properties of their films.

## 2 METHOD AND MATERIAL

### 2.1 Raw materials

The nano‐hydrogels of ground cellulose nanofibers of wood (WCNF) (with 2.7 wt%), Bacterial synthesised cellulose nanofibers (BCNF) (with 1 wt%) and Chitin nanofibers (ChNF) (with 1.5 wt%) nano‐hydrogels, were purchased from Nano Novin Polymer Co. (Iran). The concentration of these samples was adjusted to 0.5 and 1 wt% prior to experiments. Wood cellulose nano‐hydrogels have been produced with the effect of mechanical forces on pure cellulose fibres of lignocellulosic sources. During the production process using super disk mill, the diameter of the cellulosic fibre decreased from 30,000 nm to less than 50 nm. Chitin is a bio‐polymer and found in hard shells of shrimp and crab. Hereafter, WCNF, BCNF and ChNF nano‐hydrogels with the concentration of 0.5% and 1% are referred as to WCNF 0.5, WCNF 1, BCNF 0.5, BCNF 1, ChNF 0.5 and ChNF 1.

### 2.2 Nanofilm production

Nanofilms were produced with vacuum filtration method in which the Buchner funnel (with the diameter of 12 cm) and a filter flask connected to a vacuum pump were used. The nanofilms used for this study had grammage parameter (weight to area ratio) of 60 g/m^2^. Also, polyester screen with mesh size of 350 was placed at the bottom of the Buchner funnel to allow water to drain and to make a nanofiber mat on the top of the screen. The mat was then dried in a vacuum oven at 90°C for 3 h to produce nanofilms.

### 2.3 Rheological properties

Rheological properties of nano‐hydrogels have been evaluated by the Anton Paar Physica rheometer (Anton Paar, MCR301, Austria) equipped with Bob & Cup geometry (13.319 mm bob radius, 14.465 mm cup radius, 40.009 mm gap length, 120° cone angle, and 1.146 mm measuring gap).

#### 2.3.1 Steady shear measurement

For all samples, flow behaviour was estimated by enhancing the shear rate from 0.01 to 1000 S^−1^. The flow behaviour data were fitted with various rheological models, including the Newtonian model (Equation 1), Power law (Equation 2), Herschel–Bulkley (Equation 3), Bingham (Equation 4) and Cross (Equation 5) to find the best correlation between the shear rate (γ) and shear stress (τ), as shown below;

(1) τ=μ.γ

(2) $$\tau =k\gamma^{n}$$

(3) $$\tau =k\gamma^{n}+\tau {\;}_{0}$$

(4) $$\tau =\tau {\;}_{0}+\eta_{p}\gamma$$

(5) $$\eta_{a=}\eta_{\infty \;+}\frac{\eta_{0}-\eta_{\infty }}{1+{\left(\alpha_{c}\gamma \right)}^{m}}$$

where, *τ*: shear stress (Pa), γ: shear rate (s^−1^), *μ*: Newtonian viscosity, *k*: consistency coefficient (Pa.s^n^), τ_0_: yield stress (Pa), *n*: flow behaviour index (dimensionless), $\eta_{p:}\;$ Bingham plastic viscosity, $\eta_{0:}$ zero shear viscosity (Pa.s), $\eta_{\infty }:$ infinitive shear viscosity (Pa.s), α_*c*_ and λ_*c*_: time constants related to the relaxation times of the polymer in solution, *m* and *N*: dimensionless exponents [12].

#### 2.3.2 Oscillatory shear measurement

Amplitude sweep tests were done over a strain range of 0.01%–100% at a frequency of 1 Hz and 20°C, and storage modulus (G'_LVE_), viscous modulus (G″_LVE_) at the LVE (linear viscoelastic region) region and cross over point were determined [12]. Frequency sweep was done at the frequency range of 0.01–100 rad/s, strain of 0.1% and 20°C.

### 2.4 SEM

For microscopic study, the WCNF, BCNF, and ChNF films were observed using a scanning electron microscope (SEM; VEGA, TESCAN‐XMU, Czech Republic). The nanofilms were first sputter‐coated with a thin gold layer before SEM observation at 15 kV.

### 2.5 AFM

The surface topography of the untreated and surface ‐modified wooden/bacterial cellulose and chitin nanofilms were assessed using the AFM (Ara research, Full‐Model, Iran). The AFM was operated in the tapping mode with horizontal and vertical resolution of 0.26 and 0.10 nm, respectively. The values of Absolute roughness (Ra) and Root Mean Square roughness (RMS) were obtained based on an average from 5 independent measurements on regions of 5 × 5 mm [13].

### 2.6 FTIR

FTIR spectra of BCNF, ChNF, and WCNF were measured from wavenumber 400–4000 cm^−1^ by a Bruker Equinox 55 spectrometer. For BCNF, ChNF, and WCNF, samples were studied using KBr to form pellets. For every range, 16 scans at a resolution of 4 cm^−1^ were captured [14].

### 2.7 Tensile test

An all‐inclusive testing machine outfitted using a 60N load cell was used to determine tensile strength (TS), elongation at break (EAB) and elastic modulus (EM). Nanofilm strips of 110 × 20 mm were adapted at 23°C and 53% relative dampness in an environmental chamber before testing. Initial grip separation and mechanical crosshead speed were set at 50 and 5 mm/min, respectively. Nanofilm elongation at the break point (E) was calculated by Equation 6. Tensile strength shows the tolerable maximum tensile stress of any material without permanent strain. Elongation percent at breaking point shows the flexibility of the nanofilms [15].

(6) Elongationpercenttobreakingpoint=extension/(Initiallength)×100

### 2.8 Statistical analysis

The measurements were carried out in a completely randomized design. The experimental data were analysed using SPSS statistical software (Version 23, IBM, NY, U.S.). One‐way analysis of variance was used to find significant responses, where the means were compared by Duncan test (*p* < 0.05). Rheological data were analysed using Rheoplus software (RHEOPLUS/32 V3.40, Germany). The curves were drawn with Excel 2013 software. RMSE (root mean square errors) values of models were calculated with Matlab 2016a software (R2016a (9.0.0.341360), U.S.).

## 3 RESULTS

### 3.1 Flow behaviour

**FIGURE 1:**
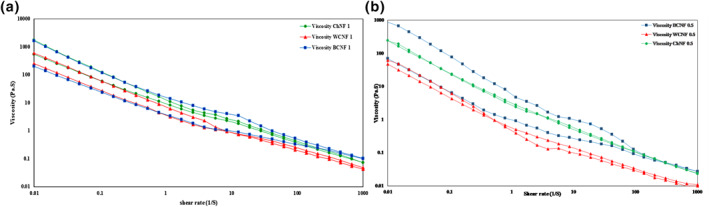
Viscosity of 0.5 wt% (b) and 1 wt% (a) concentration of WCNF, BCNF and ChNF nano‐hydrogels as a function of shear rate

The flow curves of WCNF, BCNF, and ChNF nano‐hydrogels at 0.5% and 1% concentration are shown in Figure 1. Apparent viscosity decreased as the shear rate increased in all the nano‐hydrogels indicating a pseudo‐plastic behaviour in all samples. Shear thinning behaviour of nano‐hydrogels can be due to the rupture of the weak bonds between the particles in nano‐hydrogels [16]. Similar results were reported by some researchers [3, 17, 18, 19, 20, 21, 22]. As shown in Figure 1, apparent viscosity was found to be higher in higher concentrations of nano‐hydrogels (1%w/v), probably attributing to the increase in the molecular weight, and forming a three‐dimensional structure by increasing the nano‐hydrogel concentration [23].

The results of fitting flow behaviour data calculated using different equations are indicated in Table 1. The flow behaviour data in all samples were fitted well with the Herschel‐Bulkley model with a high coefficient value and low RMS, so this model was chosen as the best model. As shown in Table 1, consistency coefficient (*k*) of ChNF (1%) was higher than other samples (1.8 Pa.sn). K value was higher in higher concentration of nano‐hydrogels, and the lowest *k* value belonged to the WCNF and B‐CNF with a concentration of 0.5% (*p* < 0.05). Higher K value can be due to more association between nano‐hydrogels and solvent molecules [24]. Flow behaviour index (*n*) of samples was in the range of 0.43–0.64 confirming the shear thinning behaviour of all samples. The highest *n* value belonged to the WCNF with a concentration of 0.5% (*p* < 0.05).

**TABLE 1:** Effect of nano‐hydrogels’ type and their concentration on the rheological parameters based on Herschel‐Bulkley, Newtonian, Ostwald, Bingham and Cross models

Nano‐hydrogels	C (%)^#^	Herschel‐Bulkley					Ostwald		Newtonian		Bingham		Cross	
		τ_0_(Pa)^##^	K(Pa.sn)***	n^###^	*R* ^2^	RMSE**	*R* ^2^	RMSE	*R* ^2^	RMSE	*R* ^2^	RMSE	*R* ^2^	RMSE
B‐CNF	0.5	1.14 ± 0.1^d^*	0.77 ± 0.1^c^	0.55 ± 0.04^ab^	0.74	3.4	0.69	3.8	0.12	6.3	0.68	0.84	0.6	4.25
	1	2.06 ± 0.08^b^	1.61 ± 0.15^a^	0.55 ± 0.01^ab^	0.9	8.3	0.89	8.7	0.47	19.7	0.77	13	0.84	5.36
W‐CNF	0.5	0.96 ± 0.03^d^	0.51 ± 0.04^c^	0.64 ± 0.06^a^	0.98	0.33	0.96	0.46	0.8	1	0.93	0.62	0.73	8.35
	1	1.62 ± 0.17^c^	1.21 ± 0.08^b^	0.45 ± 0.07^b^	0.96	2.16	0.95	4.1	0.4	9.47	0.78	5.67	0.76	7.12
ChNF	0.5	1.32 ± 0.1^cd^	1.08 ± 0.11^b^	0.43 ± 0.04^b^	0.96	3.4	0.98	0.89	0.36	4.4	0.74	9.53	0.87	3.28
	1	2.76 ± 0.23^a^	1.8 ± 0.14^a^	0.44 ± 0.06^b^	0.99	0.29	0.95	4.1	0.04	18.5	0.78	2.22	0.91	5.26

* In each column numbers relate to each variable of at a concentration of 0.5% with different letters showing significant difference (*p* ≤ 0.05) (a, b, c, d).

** Root‐Mean‐Square Error, ^##^ yield stress.

*** Consistency coefficient, ^###^ Flow behaviour index.

^#^ Concentration.

The values of yield stress calculated from the Herschel‐Bulkley model is also indicated in Table 1. The highest value of yield stress belonged to the ChNF with a concentration of 1% (2.76 Pa) while the lowest amount of this parameter belonged to the WCNF with a concentration of 0.5% (0.96 Pa).

The comparison of flow behaviour at both regions of shear rate increase (0.01 to 1000 s^−1^) and its decrease (1000 to 0.01 thixotropic behaviour). The hysteresis loop showed time‐dependent flow behaviour, and its area was used for calculation of the thixotropic value [3, 25]. The viscosity of the thixotropic fluid decreased at shear time showing the time‐dependent behaviour of the fluid (Table 2). Moreover, the thixotropic character was found to be more pronounced when cellulose concentrations increased [3].

**TABLE 2:** Results of Hystersis measurement

Nanogel	Hystersis
BCNF‐0.5%	53.79 ± 4.52^A^
BCNF‐1%	44.74 ± 4.52^A^
WCNF‐0.5%	13.7 ± 0.795^B^
WCNF‐1%	3.16 ± 0.795^B^
ChNF‐0.5%	4.7 ± 1.116^B^
ChNF‐1%	20.36 ± 1.116^B^

In each column numbers with different letters had significant difference (*P* ≤ 0.05) (A, B, C, …).

**FIGURE 2:**
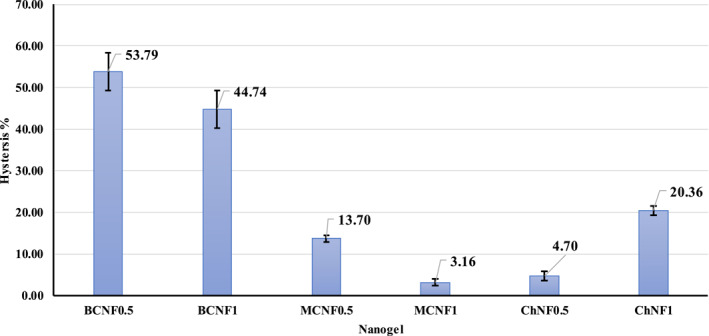
Hysteresis area of nano‐hydrogels with 0.5 and 1 wt% concentration

The hysteresis area of WCNF, BCNF, and ChNF nano‐hydrogels at concentrations of 0.5% and 1% are shown in Figure 2. BCNF nano‐hydrogels had the highest hysteresis appearance (their mean was 49.265) showing that the gel structure has been broken when the shear rate has increased, and as it decreased, the gel structure did not recover its primary structure thus, both upward and downward curves did not match. When the gel hysteresis region increase was seen, its time dependence increased [26]. WCNF nano‐hydrogels had the best structure recovery and lowest hysteresis appearance (their mean was 8.43), probably formed again due to the presence of cellulosic fibres and hydrogen bonds. ChNF (their mean was 12.408) had a very porous structure, with several nano‐roots when its concentration was increased. The gel structure was broken when the shear rate was increased, and as it decreased, the gel structure did not recover its primary structure thus both upward and downward curves did not match. As a result, the ChNF hysteresis area increased compared to WCNF and BCNF. When the gel hysteresis region increase was seen, its time dependence increased.

### 3.2 Amplitude sweep

**FIGURE 3:**
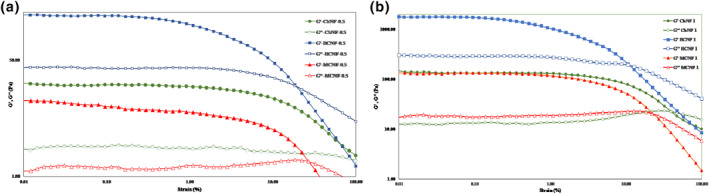
Storage and loss modulus of 0.5 wt% (a) and 1 wt% (b) concentration of WCNF, BCNF and ChNF nano‐hydrogels as a function of strain

Amplitude sweep test for nano‐hydrogels was done at 20°C and 1 HZ. LVE of nano‐hydrogels was determined using G′ and G″. At LVE, G′ and G″ had a constant value, while on increasing the strain a decrease was observed. As shown in Figures 3a,b, G′ was greater than G″ in the initial range of the strain but they crossed over each other on increasing the strain.

Rheological parameters extracted from Figure 3 are shown in Table 3. The strength of the gel sample can be described by determining G′ and G″ at LVE [27]. G′_LVE_ had a higher value at the B‐CNF nano‐hydrogel compared to other samples. The high variation of the G′ and G″ modulus shows that the formation of the nano‐hydrogel network is probably due to the strong cross‐linking [2].

**TABLE 3:** Amplitude test parameters of different nano‐hydrogels

Nano‐hydrogels	Concentration (%)	G'LVE	G″LVE	Cross over point (%)
B‐CNF	0.5	222 ± 7	38.2 ± 2.1	18.4 ± 2.3
	1	1760 ± 21	290 ± 6.9	10.6 ± 0.8
W‐CNF	0.5	11.45 ± 1.1	2.6 ± 0.3	26.8 ± 2.7
	1	130 ± 2.1	18.1 ± 0.3	22.2 ± 1.9
ChNF	0.5	21.1 ± 0.2	2.7 ± 0.1	47.2 ± 3.5
	1	129 ± 4.2	12.25 ± 0.7	39.1 ± 3.1

With an increase in the concentration, G′_LVE_ increased in all samples. The gel structure was dependent on the concentration of the nano‐hydrogel as seen by the increase in G′ and the extension of the linear viscoelastic region with concentration [28]. The lowest amount of G′_LVE_ belonged to the WCNF nano‐hydrogel with a concentration of 0.5% (11.45 Pa) and the highest value belonged to the BCNF with a concentration of 1% (1760 Pa). A similar trend was observed in G″_LVE_. G″_LVE_ was found to be highest in the BCNF with a concentration of 1% (290 Pa). A cross over was observed in the samples, in which G′ is equal to G″ at this point. At the low‐strain region before the decrease in *G*″, the used strain energy was mostly absorbed by the network structure [9]. Below the critical strain point (γc), rheological properties of viscoelastic materials are independent from strain values. After the cross over point, a change occurs in the structure and a viscose behaviour is dominated [12]. An elastic behaviour was observed before the cross over point, after which the behaviour was changed. The network structure disrupts at strains higher than the critical strain point. which in turn leads to a change in the sinusoidal form of the stress wave. Storage and loss modulus were found to be dependent to the strain amplitude [12]. By increasing the concentration of nano‐hydrogels, cross over occurred in the lower strain. The results of the amplitude sweep test showed that by increasing the concentration, the strength of the samples was improved. Also, the BCNF nano‐hydrogel had the best structure matrix. BCNF with 0.5 wt% of concentration had a higher texture strength compared to other nanofiber nano‐hydrogels. Also, the BCNF nano‐hydrogel with 1 wt% concentration had high texture strength, but the texture strengths of WCNF and ChNF nano‐hydrogels were equal. Thus, it is concluded that the stronger texture is a result of higher nano‐hydrogel concentration. Hosseini et al. [14], reported that the BCNF had a uniform structure leading to higher strength and stability of the nano‐hydrogel.

### 3.3 Frequency sweep test

**FIGURE 4:**
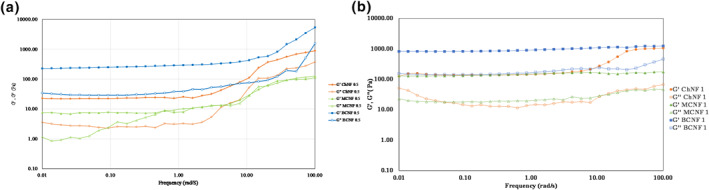
Storage and loss modulus of 0.5 wt% (a) and 1 wt% (b) concentration of WCNF, BCNF and ChNF nano‐hydrogels as a function of frequency

The mechanical spectra for different nano‐hydrogels with a concentration of 0.5% and 1% are shown in Figures 4a,b. As illustrated in Figure 4, storage modulus was greater than loss modulus in all samples during the frequency sweep test. The higher value of the storage modulus represents a solid like behaviour in all samples. [29] The storage modulus and the loss modulus increased with an increase in the concentration [3].

The results showed the storage and loss modulus increased in all samples with an increase in the frequency. These results were more obvious at a lower concentration of nano‐hydrogels. These results are in line with those of the previous studies [23]. Increase in the storage modulus following the increase in the frequency can be related to the formation of macromolecular structures at a higher frequency range [29]. Increase in the loss modulus following the increase in the frequency can be related to the rupture of the bonding for which there is no time to rebuild their structure. There was no crossover in the frequency sweep test in all samples indicating a solid‐like behaviour of nano‐hydrogels [30]. There is a higher frequency dependency in the physical gels, while a low frequency dependency represents the covalent gels [31].

As shown in Figure 4, there is a low frequency dependency in all nano‐hydrogels with a concentration of 1% while a higher dependency was observed in samples with a concentration of 0.5%. In nano‐hydrogels with a concentration of 0.5%, a higher dependency was observed at a higher frequency range attributing to the increase in the hydrogen bonding of the nanoFibers with a lower concentration and enhancement in the formation rate of the inherent network, thereby resulting in an increase in the rheological parameters [32]. Graphs had two linear and nonlinear regions at 0.5 wt% concentration. At the linear region, storage and loss modulus had quite slight changes and did not show dependency to frequency variations representing an ideal gel behaviour (G'>G″). These results are consistent with the prior studies [12, 23]. At the nonlinear regions, both the storage and loss modulus increased as a result of increasing the frequency. In the gel state, the elastic behaviour is dominated, that is, G'>G″, G' ≈ *ω*_0_, and G" and ω_0_. These relationships can be obtained from adequately high concentrations of the physical polymer network or the chemical structure of the polymer in the solvent [13]. Based on these results, nano‐hydrogels with a concentration of 1% had a higher tendency to form a stable structure [12]. The BCNF nano‐hydrogel showed the lowest frequency dependency compared to other nano‐hydrogels demonstrating a higher strength in their structure. Nanofiber nano‐hydrogels had a stronger structure and texture when their concentration increased [13] resulting from a more uniform texture and fewer pores of BCNF nano‐hydrogels, while the ChNF and WCNF nano‐hydrogels had a porous texture and lower strength [14]. Nano‐hydrogels had an elastic behaviour at the low frequency and a non‐elastic behaviour beneath the high‐frequency region. Nano‐hydrogels keep the elastic behaviour. However, due to the highly entangled and rigid matrix of nanofibers at the low‐frequency region, this matrix loses its strength and becomes multi‐phase with a viscous behaviour as a result of the increase in the G″ in the high‐frequency region [13]. The critical (yield) strain is almost independent from nanofiber nano‐hydrogels’ concentration, used to predict the apparent yield stress [17, 19].

### 3.4 SEM

**FIGURE 5:**
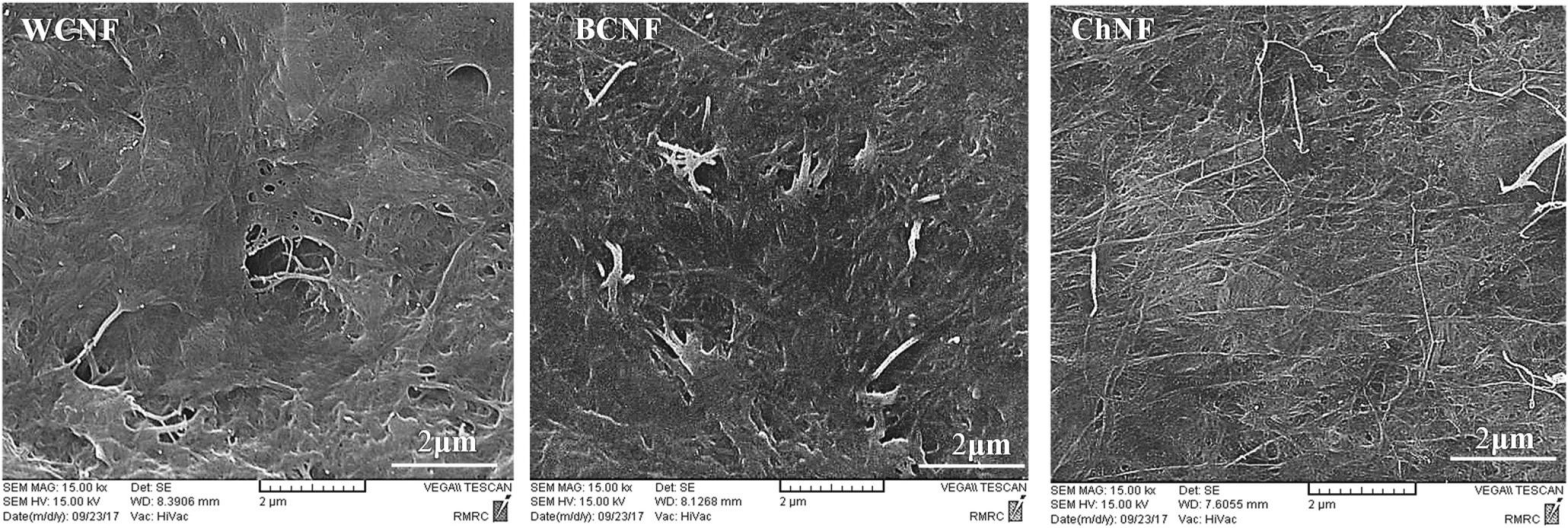
SEM images of WCNF, BCNF and ChNF

Figure 5 shows the SEM micrographs of WCNF, BCNF, and ChNF.

The thickness of the films varied between 0.0055 and 0.0085 mm and was determined using a micrometre. The thickness means (0.0079 mm) of WCNF nanofilms was higher than of other nanofilms. The average diameters of the WCNF, BCNF, and ChNF were measured as 35, 48, and 26 nm, respectively, confirming that all nanomaterials used in this study were in nanoscale (below 100 nm) with fibre morphology. Since the nanofibers have network structures, the measurement of the nanofiber length was difficult, but it can be roughly estimated as longer than 10 μm. The length of the BCNF was reported to be longer than that of the WCNF [6]. Based on the roughly estimated nanofiber length, the aspect ratio (ratio of length to diameter) can be calculated to be more than 200. The aspect ratio is one of the main parameters determining the mechanical properties of the nanofibers [33].

### 3.5 AFM

**FIGURE 6:**
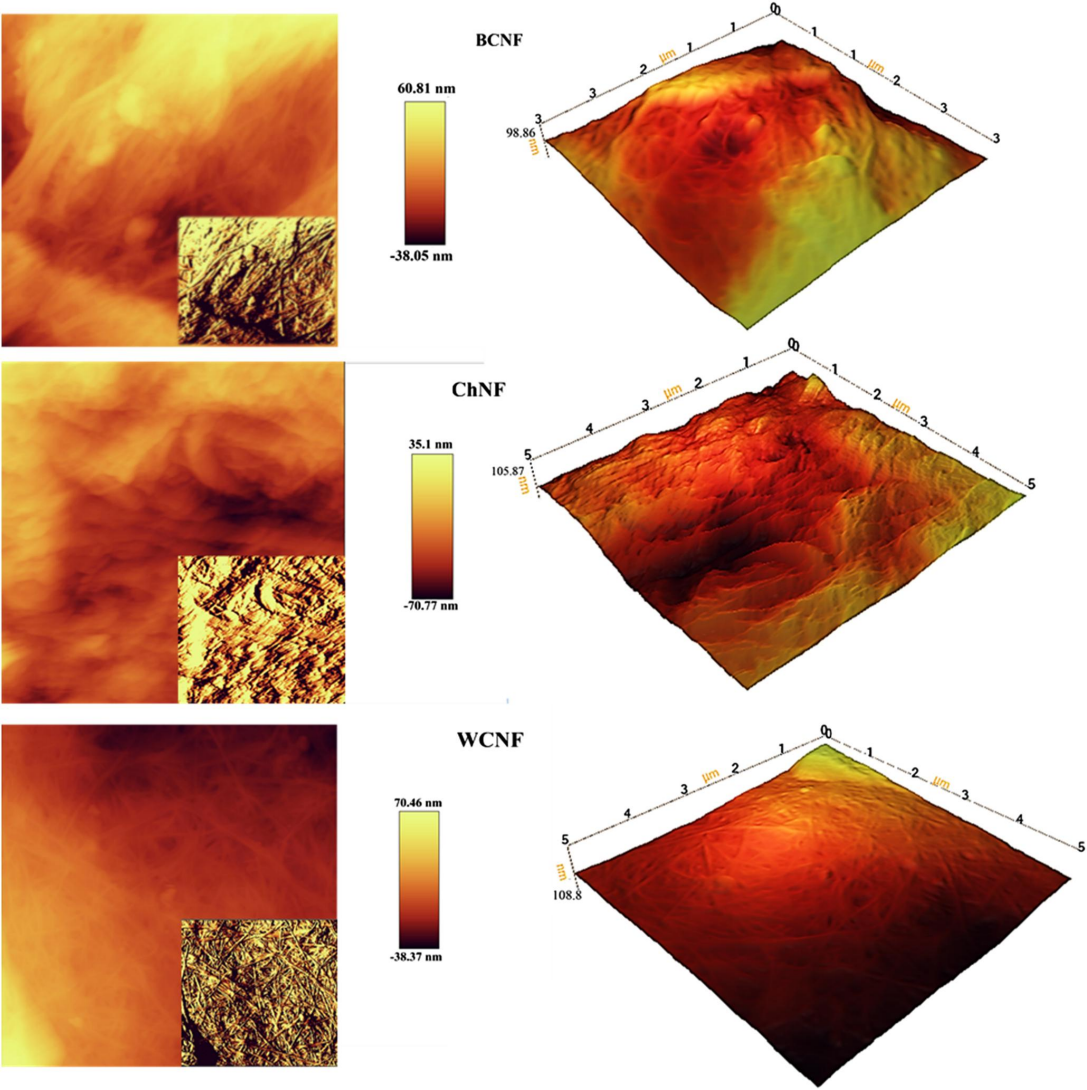
AFM topographic images of BCNF, ChNF and WCNF

The Atomic Force Microscope (AFM) is an excellent device for analysing the surface of rigid materials, providing high‐resolution topographic images and measuring surface roughness in addition to determining the accurate size and size distribution of the particles [13]. The AFM is an appropriate method to characterise nanofibers because it provides accurate information about the diameter, length and shape of the nanofibers [34]. As illustrated in Figure 6, the W‐CNF had a higher height difference (108.8 nm) resulting from the cellulosic strands arrangement and their non‐uniformity. The B‐CNF had a lower height difference (about 98.8 nm) because of its more uniform surface and structure. The diameters of the nanofilms were approximately 100 nm. Previous work [35] has reported the same results for morphological properties of nanofilms by AFM.

### 3.6 FTIR

**FIGURE 7:**
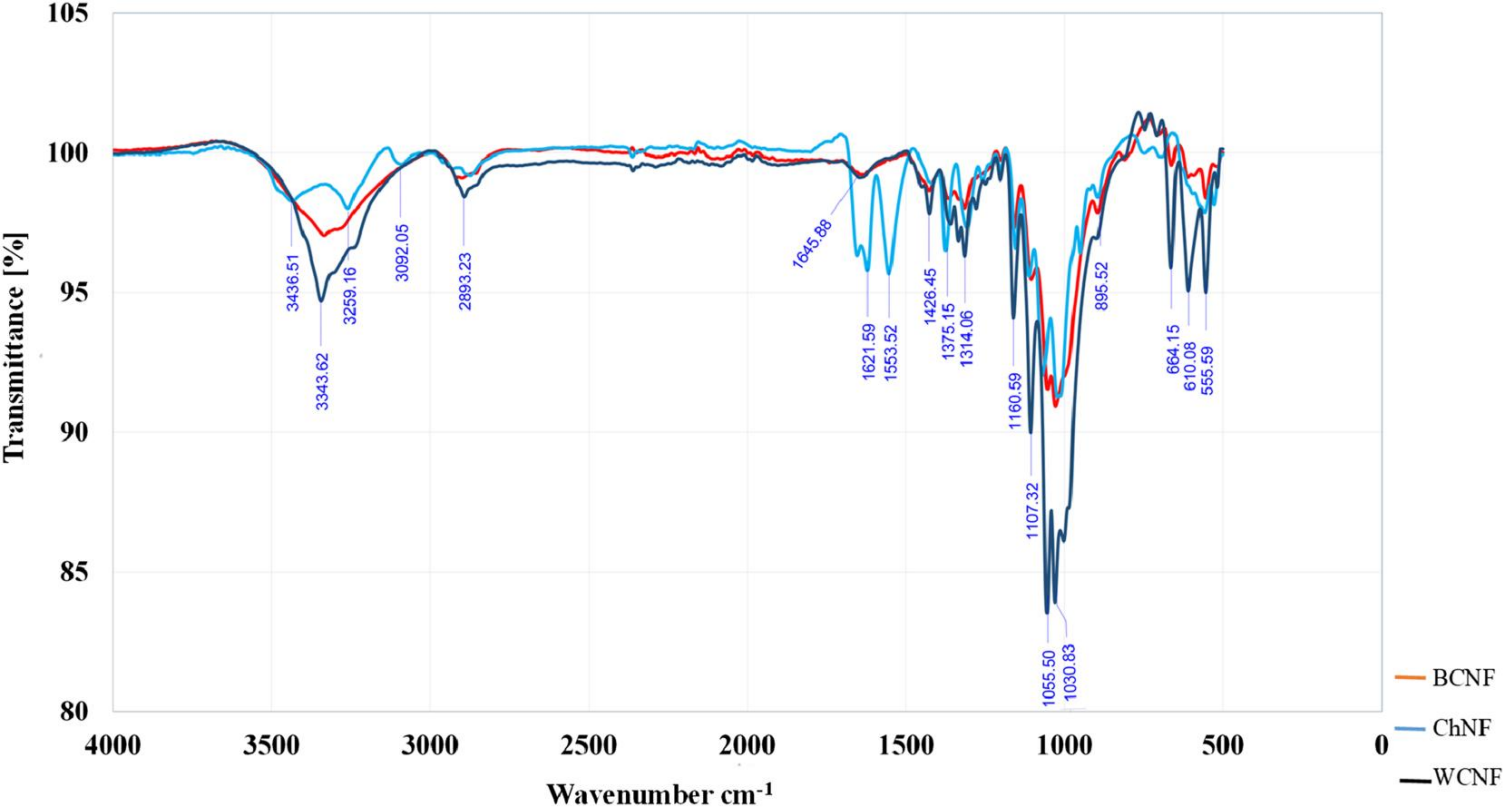
FTIR spectra of BCNF, ChNF and WCNF

The FTIR analysis was done to characterise the surface functional groups of the WCNF, BCNF, and ChNF. As depicted in Figure 7, most of the FTIR peaks are the same for BCNF, WCNF, and ChNF. Peaks at 3343 cm^−1^ (WCNF), 3334 cm^−1^ (BCNF), and 3259 cm^−1^ (ChNF) were related to carboxylic acid groups (RCO‐OH). The dominant absorption peaks at 3360 and 3320 cm^−1^ were attributed to the development of hydrogen bond groups (‐OH) [36].

The wide peaks at approximately 3340 cm^−1^ were attributed to O‐H stretching vibrations. [37] Spectral peaks observed at 3435 cm^−1^ (ChNF) were due to the stretching of ‐OH (Alcohols (RCH_2_OH)) bonds and peaks at 2900 cm^−1^ (WCNF) and 2874 cm^−1^ (BCNF, WCNF, ChNF) were due to the stretching bonds of ‐CH (Alkanes (RCH_2_CH_3_)) [38]. Various absorption peaks between 2850 and 2910 cm^−1^ showed the stretching vibration of saturated ‐CH_3_ groups [36]. An area measuring 2800–3000 cm^−1^ was associated with the stretching modes of the C‐H bonds of methyl groups [12]. The peaks which were seen at 3092 cm^−1^ (RCH = CH_2_ (=C‐H)) (ChNF), 664 cm^−1^ (Alkynes (RC = CH) (WCNF), and 610 cm^−1^(Alkynes (RC = CH) (WCNF) corresponded to the C‐H stretching vibration [39]. The characteristic peaks at 1631 cm^−1^, 1621 cm^−1^ (ChNF) were related to amides (RCONH_2_). Peaks at 1600–1750 cm^−1^ were indicative of amide ɪ and N‐H bending [12]. The peaks at 1426 cm^−1^ (WCNF) corresponded to ‐CH_2_ stretching vibrations [40]. The sharp peaks at 1375 cm^−1^ can be attributed to the CH_3_ symmetric developing band [14]. Peaks at 1200–1240 cm^−1^ (WCNF) showed the attendance of sulphate groups on the surface of cellulosic films [38]. The characteristic peaks observed at 1314, 1160, 1107, and 1030 cm^−1^ were indicative of alkyl halides (R‐F). The peaks at 1055 cm^−1^ were assigned to the pyranose ring ether band of cellulose in the WCNF. The peak at approximately 895 cm^−1^ was associated with cellulosic *ß*‐glycosidic linkages [37]. Peaks at 665 cm^−1^ corresponded to the stretching of the C‐OH bond [38]. The peaks at 610 cm^−1^ were related to Alkynes, and those seen at 555 cm^−1^ were related to Alkyl halides (R‐Br).

### 3.7 Tensile test

**FIGURE 8:**
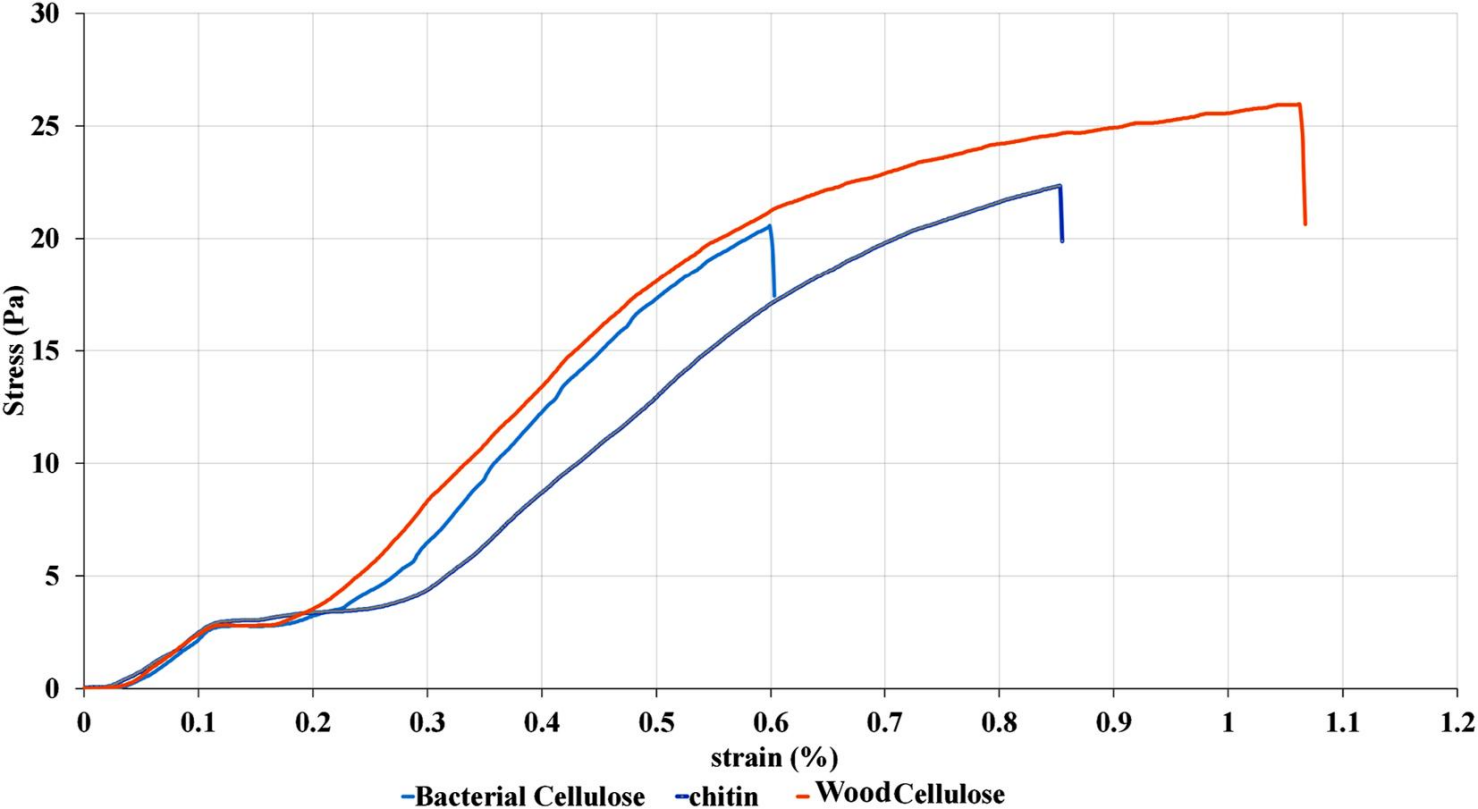
The stress‐strain curve of WCNF, BCNF and ChNF

Tensile behaviour of the nanofilms is very important in choosing different applications for polymeric formulations. Parameters as tensile strength (r), modulus (E), and elongation at break (Ɛ_*b*_) demonstrate the nanofilms’ ability to keep up the totality under stress occurrence, pending the processing, management, and the storage of the packaged materials [41]. It is very favourable that, an edible nanofilm keeps up its totality during processing, conveyance, and handling. Mechanical properties are important in the edible nanofilms, because an appropriate mechanical strength guarantees the totality of the nanofilm and its liberty from the lowly imperfections such as pinholes [14]. Figure 8 shows the stress‐strain curve of the WCNF, BCNF, and ChNF nanofilms. A drop was seen in the curves in the stress‐strain curve at the 0.1%–0.2% strain range . In the tensile test, nanofilms showed two different regions, elastic (reversible) and plastic (irreversible) regions. The region between 0.1% and 0.2% ranges of the strain is called the yield strength. In this region, a change from elastic to plastic deformation of nanofilms occurred and they could not recover their initial shape when the force was removed.

After elastic region, the plastic region was seen as force deformation increased. In this region, strain changes were almost independent from the stress changes, and at the constant stress (about 4 Pa), the strain increased from 0.1 to about 0.2%. At low strain, elastic deformation happened as a result of the nature of the cellulose fibril network followed by a macroscopic plastic yielding which were seen as a slight change in the slope. In the plastic region, the slope was straight and the strain‐hardening behaviour occurred led to fibril stretching and slight reorientation [42]. The wooden cellulose nanofilms had a lower yield strength region, and, on the contrary, chitin nanofilms showed a higher yield strength region.

The increase in the strain‐to‐failure may be related to favourable deformation of the *ß*‐sheet structure, in the unscrewing form, as far as the cellulose nanofibrils are displaced with respect to each other in the strain‐hardening region. After unscrewing, the cellulose molecules create electrostatic interactions among nanofibrils resulting in a smooth slope in the plastic deformation region [42].

#### 3.7.1 Peak force (max)

Peak force data are shown in Table 4. The WCNF nanofilms had the highest maximum tolerable force. The BCNF nanofilms had the lowest peak force. As the WCNF were produced from pine wood, they had more strength and interconnected structure, thus they could better tolerate the forces.

**TABLE 4:** Results of Nanofilms peak force measurement

Nanofilm	Peak force
WCNF	25.97 ± 4.459^A^
BCNF	20.58 ± 3.741^B^
ChNF	22.34 ± 3.858^A^

All numbers are expressed as percent (mean ± std. error).

In each column numbers with different letters had significant difference (*P* ≤ 0.05) (A, B, C, …).

#### 3.7.2 Extension

The ChNF nanofilms had the highest extension mean, while the BCNF nanofilms had the lowest extension mean. The WCNF nanofilms had extension mean nearly to the ChNF nanofilms. The ChNF and WCNF nanofilms had more stretch tolerance compared to the bacterial cellulose nanofilms.

#### 3.7.3 Stress

The ChNF nanofilms could tolerate the highest stress mean, while the WCNF and BCNF nanofilms could tolerate and had the lowest stress mean, attributing to the functional groups organised on the polymer surface enhancing the interfacial connections, outstanding to a better stress transmission among the polymer continuums and the encompassing matrix, and eventually more elongation at breaks [43].

#### 3.7.4 Elongation (strain)

Elongation data are shown in Table 5. The ChNF nanofilms had the highest elongation (strain) value, because these nanofilms tolerated the highest stress, as a result they had the highest response. The BCNF nanofilms broke at the lowest stress; hence they had the lowest elongation (strain) value.

**TABLE 5:** Results of Nanofilms elongation measurement

Nanofilm	Elongation
WCNF	3.5417 ± 1.034^A^
BCNF	1.9936 ± 0.412^A^
ChNF	2.8442 ± 0.506^A^

All numbers is expressed in percent (mean ± std. error).

In each column numbers with different letters had significant difference (*P* ≤ 0.05) (A, B, C, …).

## 4 DISCUSSION

The viscosities of hydrogels decreased monotonically with an increasing in shear rate, namely shear thinning [13, 48]. The viscosity increased when shear stress decreased, but its graph was not overlapped when the viscosity value changes with increasing shear rate. This is because the shear rate caused partial disruption of hydrogel texture. As the result of the increase in shear rate, the physical interactions between adjoining polymer chains decrease [44]. The Herschel‐Bulkley equation is preferred to power law or Bingham relationships, because it results in more accurate models of rheological behaviour when adequate experimental data are available [45]. Apparent viscosity decreased as shear rate increased in all the nano‐hydrogels indicating a pseudo‐plastic behaviour in all samples. The flow behaviour index (*n*) of samples was in the range of 0.43–0.64 confirming the shear thinning behaviour of all samples. The highest *n* value belonged to the WCNF with a concentration of 0.5%. The ChNF with 1% had the highest consistency coefficient (*k*). In return, the K value was higher at a higher concentration of nano‐hydrogels, and the lowest *k* value belonged to the WCNF and B‐CNF with a concentration of 0.5%. Higher K value can be due to increased association between nano‐hydrogels and solvent molecules. The highest value of yield stress belonged to the ChNF with a concentration of 1% (2.76 Pa) while the lowest amount of this parameter belonged to the WCNF with a concentration of 0.5%. The storage modulus (G′) values of hydrogels with the same concentrations were much higher than those of loss modulus (G″); hence, their behaviour was like an ideal gel. On comparison of Figures 2, 3, it can be concluded that with decrease in concentration, the structure strength of hydrogels decreased, and at higher strain values, the hydrogel structure showed faster breakdown [27]. By increasing the strain higher than the critical strain point, the network structure disrupts and the form of stress wave exits from the sinusoidal mode. Storage and loss modulus were dependent on strain amplitude [46]. Nanofibers hydrogel behaviour was closer to liquid one and by decreasing the modulus, G″ values passed from G'. BCNF with 0.5 wt% concentration had higher texture strength compared to the other nanofiber hydrogels. Also, BCNF hydrogel with 1 wt% concentration had high texture strength, but MCNF and ChNF hydrogels are equally strong. It can be concluded that the hydrogel displayed stronger texture on increasing the concentration, . At 1 wt% concentration, B‐CNF hydrogel had lower modulus variation in frequency at a constant strain and was almost independent to frequency variation, while this nanofiber hydrogel at 0.5 wt% concentration had a critical frequency point which appeared after the linear region, where the hydrogel state be changed and the storage modulus decreased. Nanofiber hydrogels had stronger structure and texture when their concentration was increased [47]. Mechanical WM‐CNF and B‐CNF and nanofiber hydrogels at 1 wt% concentration showed similar behaviour, but the elastic modulus increased after over a critical frequency point in the other nanofiber hydrogel concentrations. Structure nanofiber hydrogels have been stronger when frequency was increased. B‐CNF had highest storage modulus and gel strength, but this hydrogel at 0.5% concentration had a weaker structure. Hydrogels had an elastic behaviour at a low frequency and a non‐elastic behaviour under a high‐frequency region. Hydrogels keep the elastic behaviour. This is because of highly entangled and rigid matrix of nanofibers at the low‐frequency region, but this matrix loses its strength and turns to be multi‐phase with a viscous behaviour because G″ was increased in the high‐frequency region [47]. The critical (yield) strain is almost independent of nanofiber gel concentration, which is used to predict the apparent yield stress [13, 48].

## 5 CONCLUSION

The results showed that all the nano‐hydrogels indicated a pseudo‐plastic behaviour in all samples. Apparent viscosity was higher in higher concentrations of nano‐hydrogels. The BCNF nano‐hydrogels had the highest hysteresis appearance. The WCNF nano‐hydrogels had the best structure recovery and lowest hysteresis appearance. Storage modulus was greater than loss modulus in all samples during the frequency sweep test. Nano‐hydrogels had an elastic behaviour at a low frequency and a non‐elastic behaviour beneath the high‐frequency region. SEM showed that all nanomaterials used in this study were in nanoscale (below 100 nm) with fibre morphology. Most of the characteristic peaks of the BCNF, WCNF, and ChNF in the FTIR test were observed in the same positions. In the tensile test, the nanofilms showed two different regions, elastic (reversible)(at low strain) and plastic (irreversible) regions. Chitin nanofilms could tolerate the highest stress mean and had the highest elongation (strain).

## CONFLICT OF INTEREST

All authors have participated in (a) conception and design, or analysis and interpretation of the data; (b) drafting the article or revising it critically for important intellectual content; and (c) approval of the final version. This manuscript has not been submitted to, nor is under review at, another journal or other publishing venue. The authors have no affiliation with any organization with a direct or indirect financial interest in the subject matter discussed in the manuscript.

## Data Availability

Data available on request due to privacy/ethical restrictions.
